# A review of studies of parent-child communication about sexuality and HIV/AIDS in sub-Saharan Africa

**DOI:** 10.1186/1742-4755-8-25

**Published:** 2011-09-24

**Authors:** S Bastien, LJ Kajula, WW Muhwezi

**Affiliations:** 1 Institute for Basic Medical Sciences, Faculty of Medicine, University of Oslo, Norway, Postbox 1046 Blindern, 0317 OSLO, Norway; 2 Department of Psychiatry and Mental Health, Muhimbili University of Health and Allied Sciences, Dar es Salaam, Tanzania; 3 School of Medicine, Department of Psychiatry, Makerere University College of Health Sciences, P. O Box 7072, Kampala, Uganda

**Keywords:** sexuality communication, parent-child communication, adolescent sexuality, Africa

## Abstract

Parent-child sexuality communication has been identified as a protective factor for adolescent sexual and reproductive health, including HIV infection. The available literature on this topic in sub-Saharan Africa is increasing; however a systematic review of studies has not been conducted. This article reviews the literature in the area of parental or caregiver and child communication about sexuality and HIV/AIDS in sub-Saharan Africa. A review of peer reviewed literature published between 1980 and April 2011 was conducted. Communication process studies investigating the frequency, content, style, tone of discussions, preferences, as well as associations with and barriers to sexuality communication are reviewed. In addition, studies which examine behavioral associations with parent-child sexuality communication, and intervention studies to improve parent-child sexuality communication are examined. The findings from process studies suggest wide variation in terms of frequency of discussions, with a range of socio-demographic and other factors associated with sexuality communication. Overall, findings demonstrate that discussions tend to be authoritarian and uni-directional, characterized by vague warnings rather than direct, open discussion. Moreover, parents and young people report a number of barriers to open dialogue, including lack of knowledge and skills, as well as cultural norms and taboos. Findings are less clear when it comes to associations between parental communication and adolescent sexual activity and contraception use. However, nascent indications from intervention research suggest positive findings with increases in frequency and comfort of discussions, among other outcomes. Gaps in the research are identified and discussed with implications for future studies.

## Introduction

Improving the sexual and reproductive health of young people is a global priority. Interventions which aim to promote healthy sexual behavior typically aim to delay sexual debut, decrease the number of sexual partners and increase condom use. In spite of concerns that sexuality education may contribute to early sexual experimentation among young people, this is not supported by evidence [1]. On the contrary, findings from studies conducted in developing countries suggest that sexuality education has the potential to positively impact knowledge, attitudes, norms and intentions, although sexual behavior change has been more limited [2,3].

Adolescent sexual decision making and behavior are influenced by myriad factors at the individual level as well as peer, family, community and societal levels. Parents in particular play a substantial role in the gender and sexual socialization of their children. Discussing topics related to sexuality has been associated with a range of important psychosocial attributes including increased knowledge, better interpersonal communication skills, including sexual negotiation skills, and self-efficacy [4-8]. Communication about sexuality between parents or caregivers and offspring has also been identified as a protective factor for a range of sexual behaviors, including a delayed sexual debut, particular for females [9].

Studies focusing on parent-child communication have focused on a range of processes that may influence effectiveness in decreasing sexual risk behavior among young people such as frequency of discussions and perceptions of quality and comfort of communication [10,11]. The timing of communication is also of importance and is most likely to be effective prior to sexual debut to reinforce protective factors, but may also facilitate behavior change in those already sexually active [12]. The content of the message and how it is transmitted have also been identified as being particularly important [13]. Some studies have investigated the perceptions of young people or parents separately, whilst other studies have sought to examine differences in self-reports [10,13-16]. Growing up in a single parent household and other parental characteristics such as level of education and socio-economic status have also been explored to determine how these factors affect both communication processes and behavioral outcomes [17,18]. However, the vast majority of these studies have been conducted in North America, Europe and Australia.

Whilst programs and the literature on parent or caregiver-child sexuality communication in developing countries and sub-Saharan Africa (SSA) are limited, they are increasing [19]. This is due both to more ecological understandings of social and contextual influences on young people's sexual behavior and the need to develop more comprehensive HIV/AIDS prevention programs that include social level influences.

The role of parents in the sexual and reproductive health of adolescents in sub-Saharan Africa has been studied as part of broader efforts to understand sexual socialization and sexual agency [20,21], to determine the impact of growing up in a single or double parent household on sexual transition and sexual behavior [22-25] and to determine the influence of parental characteristics on sexual behavior [26], including social resources or parental investment [27].

Historically, the taboo nature of sexuality discussions between adults, in particular parents, and young people in sub-Saharan Africa has been well documented [28-30]. In several countries in sub-Saharan Africa, direct parental involvement in the sexual socialization of children in the past has been described as minimal. Rather, the extended family including grandparents and aunts were instrumental for imparting the necessary knowledge and skills relevant for sexual relationships [31]. With increased urbanization and social change processes however, the family unit and consequently adolescent socialization may be impacted.

The purpose of this article is to review the available literature on parent or caregiver-child communication about sexuality in SSA. In particular, we aim to review studies which focus on i) the process of parent-child sexuality communication; ii) behavioral outcomes associated with parent-child sexuality communication, and iii) interventions that have been evaluated to improve sexuality communication between parents and their children in SSA. Based on these findings, gaps for future research will be identified.

## Methods

We conducted a review of studies by searching the following databases as of April 29th, 2011: Education Resources Information Center (ERIC), PubMed, PsychINFO (Ovid), and Medline. A combination of search terms were used, including 'adolescent', 'youth', 'child', 'parent-child communication', 'family', 'caregiver', 'sexuality', 'sexual and reproductive health', 'HIV/AIDS', 'Africa' and 'sub-Saharan Africa'. In addition, a hand search of all articles identified was performed to locate other relevant studies. Inclusion for the review was restricted to those studies that were: a) published in a peer reviewed journal, b) published between 1980 and April 2011, c) conducted in sub-Saharan Africa, and d) based on empirical research concerning parent or caregiver communication about sexuality with children as a study aim or objective. We identified a total of 23 articles that met the inclusion criteria. The studies were reviewed and grouped according to whether they reported data which related to the process of sexuality communication (frequency, content, triggers, factors associated with communication, communication style and tone of discussions, preferences and barriers), which related to behavioral outcomes associated with sexuality communication (sexual debut, condom or contraceptive use) or which reported intervention data related to improving parent-child sexuality communication. These categories were subsequently used to structure the presentation of the results. Several studies presented findings which fit into both categories.

## Results

An overview of the studies included in this review can be found in Table 1. Studies were published in a range of international and African journals. The majority of studies (19 of 23) collected quantitative data. Of the quantitative studies, 14 were cross-sectional, while 5 were randomized controlled trials. Two of the papers in this review use data from the same randomized controlled trial, however the samples differ [32,33]. Only 2 studies utilized purely qualitative methods, while 2 studies used mixed methods. Of the few studies which specified a theoretical framework for the research, the Theory of Triadic Influence, Ecological Systems Theory and Social Learning Theory, were described as influential.

**Table 1:** Parent-child sexuality communication studies from sub-Saharan Africa; 1990-2011

Authors	Country	Objective	Design & population	Main findings
Adeyemo & Brieger, 1994 [42]	Nigeria (urban)	To examine discussions within the family of family life education topics (human development, sexual relationships, preparation for parenthood, pregnancy, contraception and STIs).	Parents from a total of 253 families were interviewed using a questionnaire. Systematic sampling of every sixth residential building and balloting was used to recruit families.	Parents reported discussing on average 3 out of the 6 family life education topics. Human growth and development was most frequently discussed, followed by pregnancy and childbirth and abortion. Half had discussed STIs, contraception was least discussed. Only 16% of parents had discussed all six topics. Parents did not feel competent to discuss the topics and thought it might encourage risk behavior. Mothers were most frequent initiators of discussions. Frequency of communication increased with higher level of education of the parents. Greater time spent at home and a positive perception of parental role in family life education was also associated with higher scores.

Adu-Mireku, 2003 [34]	Ghana (urban)	To evaluate the relationship between family communication about HIV/AIDS, sexual activity and condom use among secondary school students.	Cross-sectional study of 894 students in two randomly selected secondary schools.	It was reported that 25% of participants were sexually experienced. A large proportion (74%) reported having discussed HIV/AIDS with parents or other family members. Student-family communication about AIDS was not associated with sexual activity; however it did increase the odds of condom use at last sex. Significant gender differences in sexual activity, condom use and family communication about HIV/AIDS were also reported.

Amoran, Anadeko & Adeniyi, 2005 [47]	Nigeria (urban)	To determine parental role on adolescents' sexual initiation practice in Ibadan, Nigeria.	A random sample of 274 adolescents was selected from the community to complete a self-administered questionnaire on demographics and sexual behavior.	Early exposure to sex education by mothers was reported to encourage early sexual debut (p < 0.001). A greater proportion of adolescents (43%) who received sexuality information from peers were sexually experienced compared with 25.2% who sought information from parents and other sources (p = 0.004). Mothers communicated about sexuality more frequently than fathers (41% vs. 17%)..

Babalola, Tambashe & Vondrasek, 2005 [39]	Ivory Coast (urban)	To examine the relation between parental factors (living in same household as father, perceived parental disapproval and parent-child communication) and sexual risk-taking among young people	Cross-sectional household survey of randomly selected youth aged 15-24 years in three cities.	Living in the same household as father during childhood, perceived parental disapproval of premarital pregnancy and parent-child communication about sexual abstinence were positively associated with both primary and secondary sexual abstinence and a reduction in number of sexual partners.

Bastien et al., 2008 [37]	Tanzania (urban & rural)	To investigate changes in primary school students' exposure to AIDS information and communication from 1992-2005.	Repeated cross-sectional design. Students from 18 randomly selected primary schools in 2 regions completed a questionnaire (2026 students in 1992 and 2069 students in 2005).	Students reported higher levels of exposure to information and communication from all sources in 2005 than 1992. Students reported significantly more frequent discussion about AIDS with parents and others in the social network in 2005 in comparison to 1992, yet ranked low in both years in terms of who young people discussed AIDS with most frequently. Myths related to HIV transmission and infection was reported to persist.

Bastien et al., 2009 [38]	Tanzania (urban & rural)	To investigate whether young people in- and out-of-school differed in exposure to AIDS information and communication, the perceived credibility of the sources and preferences for SRH information.	Cross-sectional studies of 993 out-of-school youth and 1007 primary and secondary school students between ages of 13 and 18.	Communication about HIV and AIDs was reported to be influenced by a host of factors including access, agency and ambiguity. The in-school youth communicated about AIDS more frequently with all members of the social network investigated than out-of-school youth. Factors associated with frequency of communication included urban/rural residence, sex, age and SES. The in-school group ranked parents in the top 3 in terms of credibility and both groups identified parents as one of the top 3 preferred communicators of sexual and reproductive health information. However, both in- and out-of-school youth demonstrated an inability to critically evaluate modes of transmission and prevention.

Bell et al., 2008 [52]	South Africa (peri-urban)	To test the effectiveness of the CHAMP intervention (designed to target HIV risk behaviors by strengthening family relationship processes as well as peer influences) among black South Africans in KwaZulu-Natal.	Randomized controlled trial among youth (aged 9-13) and their families (245 intervention families rearing 281 children, and 233 control families rearing 298 children).	Findings suggest greater intervention effects on caregivers than on youth. Caregiver findings show significant intervention group differences in comparison to control with regard to caregiver monitoring and control, as well as increased frequency and comfort discussing HIV/AIDS and sexuality with children, among other outcomes. With regard to youth, findings showed an increase in HIV knowledge in the intervention group compared to the control, as well as lower levels of HIV-related stigma.

Bhana et al., 2004 [49]	South Africa (urban & semi-rural)	To prevent HIV infection in youth by promoting resiliency in pre-adolescents and their families, and strengthen community protective shield through the CHAMP (*Amaqhawe) *programme.	Experimental design with pre and post-tests. Pilot study conducted in three areas, on urban shack settlement and two semi-rural areas. A total of 124 families, assigned to intervention or comparison group participated in the pilot phase as described in this article.	In terms of parenting communication styles, the intervention group reportedly demonstrated a shift from passive aggressive and manipulative communication styles to more assertive styles in relation to comparison group. The intervention group also showed significant improvement in their ability to engage in discussion about difficult or sensitive topics with their children in comparison to control group. Frequency of discussion also improved in the intervention group, discussion about puberty increased from 55% to 69%, whilst discussion about sex which was ranked most difficult to talk about improved from 55% to 73% post intervention.

Biddlecom, Awusabo-Asare & Bankole, 2009 [41]	Burkina Faso, Ghana, Malawi & Uganda (urban & rural)	To examine the influence of three dimensions of parenting-material support (co-residence), monitoring and communication on adolescent sexual behavior.	National surveys of 15-19 year olds in each of the countries (2948 youth in Burkina Faso, 2426 in Ghana, 2025 in Malawi and 2363 in Uganda) as part of a research project called 'Protecting the Next Generation: Understanding HIV Risk Among Youth'. Two-stage, cluster design was used.	Parental monitoring was reported to be moderate to high in all countries, with females reporting higher levels of monitoring than boys. Communication about sexuality was low across countries. Between 8% and 38% of adolescents reported discussing sex with a parent figure, with even less reporting discussing contraceptive methods. Males were less likely than females to report communication. Findings were mixed in regards to parental communication and odds of sexual activity, among Malawian males and Ugandan females only, communication was associated with increased odds of having had sex in the last year (2.2 and 1.5, respectively). Parent-child sexuality communication was positively associated with contraceptive use for Ghanaian females (3.0) and Ugandan females and males (2.9 and 1.9).

Izugbara 2008 [44]	Nigeria (rural)	To explore how and why parents in rural Nigeria discuss sexuality related matters with their adolescent children.	In-depth interviews with 187 parents.	Parent-child discussions about sexuality are not common in rural Nigeria where it remains taboo to do so. Parents tend to portray sexuality as 'dangerous, unpleasant, and unsavory' in discussions with their children and tended to use threats and indirect speech in discussions. Parents worried that discussions would encourage early sexual experimentation.

Karim et al., 2003 [46]	Ghana (urban & rural)	To identify factors associated with elevated risks of pregnancy and sexually transmitted infection among unmarried Ghanaian youth.	Cross-sectional survey of a nationally representative sample of 3,739 unmarried 12-24-year-olds.	Among male youth in the study, communication about avoiding sex was found to be associated with a lower probability of having had sex (OR = 0.87), while communication about contraceptive use was associated with an increased likelihood of being sexually experienced, among both sexes (OR = 1.25 & OR = 1.23 for males and females, respectively). The association between communication about sex and contraception and condom use was overall weak, except where it concerned consistent condom use with the last partner among males (OR = 1.05). It was also found that communication about contraceptives was associated with a decreased likelihood of condom use at first sex among males (OR = 0.87).

Kawai **e**t al., 2008 [33]	Tanzania (urban)	To examine parents' and teachers' communication about sexuality in relation to timing of sexual debut among students.	Randomized controlled trial (only data from first and second post intervention follow up reported) focused on promoting sexual and reproductive health of primary school students. 2477 virgin primary school students were followed prospectively for 6 months to investigate sexual initiation and completed the questionnaire.	27% of students reported communicating about HIV and sex with parents, but parental communication was not associated with delay of sexual debut, whereas communicating with teachers was. Those not living with their biological mother were more likely to initiate sex than those who do.

Kumi-Kyereme et al., 2007 [45]	Ghana (urban & rural)	To examine connectedness, communication and monitoring of unmarried adolescents by parents, other adults, friends and social institutions, as well as the roles these groups play in influencing adolescent sexual activity.	Uses nationally-representative survey data of adolescents aged 12 to 19 years in addition to focus group discussions (14-19 years) and in-depth interviews with adolescents (12-19 years).	High levels of connectedness to family were reported in addition to high levels of monitoring. Less frequent communication with family about sex-related matters compared to non-family members. Sexuality communication was reported to be more common among older respondents than younger ones. Both males and females reported more sexuality communication with mothers than fathers. There was a strong negative relationship between parental monitoring and recent sexual activity for males and females. In terms of communication, findings show that discussing with fathers has a negative association with sexual activity for males (OR = 0.46), whilst it is positive for females (OR = 1.76). Focus group discussions point to intergenerational challenges of communicating about sexuality in the home, with some young people feeling too shy to discuss sexuality with parents and fearing physical punishment for doing so. Interviews found that when communication did take place it was likely to take the form of instructions rather than dialogue.

Löfgren et al., 2009 [58]	Uganda (urban)	To explore how young people perceive and experience the role of parents in preventing the HIV among youth.	Qualitative study using semi-structured face-to-face interviews with 16 school going youth (8 males, 8 females) in 3 secondary schools between the ages of 18 and 20 years.	Young people perceived parenting styles as negatively influencing HIV prevention among youths, for instance they felt that parents tend to use fear as a tactic in discussing about sex, and also that parents lack time to engage with their children, and when they do, they use authoritarian or indulgent parenting styles. Young people described differential treatment of boys and girls by parents.

Mathew, Shugaba & Ogala, 2006 [36]	Nigeria (urban)	To assess parent-adolescent communication about HIV/AIDS and identify factors influencing communication.	Cross sectional study of 459 junior secondary school students between 10 and 14 years of age.	Only 27% and 5% of sexually active students reported communication with parents about AIDS and sex, respectively. Inadequate knowledge on part of parents was identified as a barrier to communication by respondents (64% perceived mothers as lacking knowledge, 87% thought fathers lacked knowledge). Moreover, 62% thought that their parents are too preoccupied to talk about sex, while 59% believed their parents would argue if they were to talk about sex. 30% thought their mother would think they were interested in experimenting with sex if they were to talk about it, whilst 69% believed their father would get this impression.

Mbugua, 2007 [29]	Kenya (urban)	To examine why educated mothers in Kenya have difficulty providing meaningful sex education to their daughters.	Qualitative data drawn from 2 studies conducted in 1996 and 2003 including 4 focus groups with female secondary students aged 17-19, interviews with 14 teachers and 15 mothers.	Most educated mothers reported experiencing socio-cultural and religious inhibitions which make it challenging to provide sex-education to their daughters. For instance, generational barriers to discussing sexuality were raised as an issue. Mothers also reported a reliance on the school system to provide sex education.

Musa, Akande, Salaudeen, Soladoye, 2008 [35]	Nigeria (urban)	To examine the practice of family communication on HIV/AIDS among secondary school students.	Cross-sectional survey of 420 randomly selected secondary school students.	A large proportion (80%) of students reported family communication about HIV/AIDS, while 34% of respondents reported discussion about premarital sex with a family member. Although the study specified that the member of the family most often involved in sexuality discussions was the mother (44%), compared to the father (29%). More frequent communication was reported by females, those from smaller families and among those whose parents have higher levels of education.

Namisi et al., 2009 [32]	Tanzania and South Africa (urban & rural)	To describe adolescent sexuality communication with parents/guardians, other family members and teachers about HIV/AIDS, abstinence and condoms.	Baseline data from a cluster randomized controlled trial at 3 study sites in Tanzania and South Africa and involving 80 public schools in total and 14 944 students between 11 and 17 years. The aim was to promote the sexual health of school students and prevent HIV. Main outcome variables were frequency of communication about HIV/AIDS, abstinence and condoms.	Females preferred receiving sexuality information from their mothers, males preferred fathers. 37%, 41% and 29% reported never or hardly ever communicating about sexuality with parents, other family members and teachers. Students from Tanzania reported experiencing more silence from the various sources than in South Africa. Odds of infrequent or never communicating with parents was higher among girls than boys in Tanzania, while the reverse was true in both of the South African sites and socioeconomic status was positively associated with sexuality communication with parents.

Opara, Eke & Akane, 2010 [43]	Nigeria (urban)	To determine mothers' perception of sexuality education in children.	Convenience sample of women attending a Christian women's convention. 158 women filled in a structured questionnaire.	The majority (77%) believed that the home was the best place for sexuality education to take place with 70% believing that it is the responsibility of both parents whereas 38% thought it was the sole responsibility of the mother. In terms of content of sexuality education, discussion centered on descriptions of body parts and reproductive organs. Regarding timing of discussions of sexuality, 41% believed it should take place between the ages of 6-10 years while 32% thought it should take place between 11- 15 years. 65%) reported having discussed sexuality topics with their children, but only 12% had discussions as frequently as three times per month.

Phetla et al., 2008 [48]	South Africa (rural)	To prevent HIV and IPV by actively challenging barriers to engaging with young people about sexuality and promoting communication between adults and young people	Community cluster randomized controlled trial. Data collected at baseline and 2 years later in 4 intervention and 4 control villages. Women from poorest half of households eligible to participate, organized into 'loan centers' and 'solidarity groups'. -trained facilitators conducted interactive education and empowerment activities to raise awareness and discussion of gender inequality, IPV, sexual health and role of culture in shaping norms (program called Sisters for Life) -Four centers (120 women) were monitored to assess effects, meetings were observed and data collected using flip charts, standardized forms to record process and content of discussions. Focus groups conducted with 8 loan groups both after the intervention and one year later. Among young people, 24 interactive workshops and 6 follow-up interviews to examine perceptions of intervention and household and community effects.	Both qualitative and quantitative findings indicate the intervention improved participants' motivation and skills to engage with young people about sexuality. Interviews indicated women felt greater confidence to talk to children, used clearer messages instead of vague ones, and a range of communicative strategies. Quantitative data showed a significant increase in proportion of women at follow-up reporting having talked about sexual issues with children compared to the control group (80% vs. 49%, adjusted risk ratio 1.59 (1.31-1.93). Young people confirmed that mothers and relatives altered their communication style and content after exposure to the intervention.

Poulsen et al., 2010 [40]	United States and Kenya (rural)	To identify and compare factors associated with parent-child communication about HIV/AIDS in US and Kenya using baseline data from HIV prevention intervention called Parents Matter! In the US and Families Matter! In Kenya.	A total of 403 parent-child dyads completed the survey at baseline, but article focuses on baseline data from parents. Main outcome was whether or not parent-child communication about HIV/AIDS had occurred and to identify variables associated with communication. Participants were adults who are primary caregiver to a child aged 10-12 years at baseline and lived with the child for past 3 years.	40% of parents in Kenya had never talked to their child about HIV/AIDS, with 38% of parents thought that talking about sexuality encourages sex, and 61% believing their child was too young to learn about sex. Communication was associated with parental perceptions of child readiness to learn about sexuality, if they had received information to educate their child about sex and if they had greater sexual communication responsiveness (skill, comfort & confidence).

Vandenhoudt et al., 2010 [53]	United States and Kenya (rural)	To assess community acceptability and impact of Families Matter! Intervention on parenting practices and effective parent-child communication.	Data collected from 403 parent-child dyads at baseline and 321 parents-child dyads at 1 year post intervention. Only follow-up data reported.	High attendance from parents at all intervention sessions and reported being satisfied with the intervention, finding it helpful and a confidence booster. The majority also reported having shared intervention information with persons other than their child, indicating high levels of dissemination. Significant improvement in parental attitudes concerning sexuality education, with parents reporting greater use of positive reinforcement and monitoring. Improvements in sexual risk reduction communication, with increase from 17% to 38% baseline to follow-up of children reporting ever having asked their parent a question about sexuality, similar change among parents reported (14% to 50% at follow-up).

Wamoyi et al., 2010 [55]	Tanzania (rural)	To explore parent-child communication about sexuality in families, including the content, timing and reasons for communication with children aged 14-24 years.	Ethnographic design. Eight weeks of participant observation, 17 focus group discussions and 46 in-depth interviews were conducted with young people aged 14-24 years and parents of those in this age group.	Parent-child communication about sexuality was common in families and mainly on same sex basis. It typically consisted of warnings, threats and physical discipline and was triggered by seeing or hearing something a parent perceived as a negative experience (such as a death attributable to HIV and unmarried young person's pregnancy). A lack of trust in what they could say to their parents was reported by young people for fear of punishment. Parents were constrained in their communication due to lack of knowledge and restrictive gender and cultural norms.

The studies were conducted both urban and rural areas in East, Southern and West Africa. Participants were recruited both from school settings and within the community. The majority of studies investigated young people's perceptions (17 of 23 studies) and the age range of these studies ranged from 10 to 24 years. Ten studies incorporated data from parents, either both parents or mothers only. Three studies were cross-national comparisons, two of which focused on African countries, while the other compared young people in Kenya and the United States. The findings below are organized according to the three review objectives relating to studies focused on the process of parent-child sexuality communication, studies which investigated behavioral associations with parent-child sexuality communication and interventions which have specifically sought to improve parent-child sexuality communication.

### Studies investigating the process of parent-child sexuality communication

Communication process studies are those which describe the frequency and topic or content of discussions, triggers for discussion, the factors associated with sexuality communication, perceptions of the communication style and overall tone of discussions, preferences and barriers to communication.

#### Frequency and content of discussions

A total of 20 of 23 studies investigated frequency of discussion about a range of topics related to HIV/AIDS and sexuality between caregivers or parents and children. Firstly, in relation to HIV/AIDS, studies relying on data collected from young people reported proportions in assessing frequency of communication about HIV/AIDS with parents ranging from 8% to 80%, while other studies reported mean values [32-41]. One study looked at change over time from 1992 to 2005 in the same randomly selected primary schools in two regions in Tanzania in talking about AIDS with parents [37]. This study found that communication with parents about AIDS increased in 2005. However, as with several other studies in this review, this study did not differentiate between communication with mothers and fathers and due to the study design, it was not possible to explain the changes or attribute them to any specific intervention.

In addition to HIV/AIDS, studies investigated discussion of other topics related to sexuality. A multi-site study which assessed communication with parents about condoms and abstinence in addition to HIV/AIDS found communication on all topics was generally low and that silence was greatest on the topic of condoms [32]. This is consistent with findings from Nigeria that of 6 topics related to sexuality (development & growth, pregnancy & childbirth, preparation for adulthood, sexually transmitted diseases, contraception and abortion), contraception was least discussed [42]. Another study from Nigeria reported that 65% of mothers reported discussing 'sexuality issues' with their children at some point, however details on the recall time period and content of those discussions was not reported [43]. Also from Nigeria, one study reported that 39% of parents reported discussing sex with their child in the past year [44]. Similar findings from Kenya indicate that while abstinence, unplanned pregnancy and HIV/AIDS were topics of discussion in many families, topics that were rarely discussed include the use of contraceptives and condoms. This was attributed to a number of reasons such as parental fears concerning potential side effects such as infertility, that it would contradict their intended message emphasizing abstinence and due to shyness and lack of knowledge.

This is in contrast to a study in Nigeria in which 98% of students reported discussion about condoms with a 'family member' [35]. This study also found that 34% of respondents reported discussion about premarital sex with a family member. Although the study specified that the member of the family most often involved in sexuality discussions was the mother (44%), compared to the father (29%), the study did not make clear which family member was involved in discussions for each topic investigated. Another study which specified which family member was communicated with (but which did not specify which topic) found that the mother (with 33% of female adolescents and 16% of males) was the most frequently reported person with whom adolescents discussed sex-related matters, in contrast to the father (13% of females and 12% of males) [45]. One study in Ghana assessed whether or not communication about avoiding or delaying sex took place in the last year and also whether they had discussed the use of modern contraceptives to prevent unintended pregnancy, with the mother or female guardian, father or male guardian, aunt, uncle or sibling and found that communication about these topics is low [46]. A study conducted in Burkina Faso, Ghana, Malawi and Uganda found that whilst the proportion of adolescents reporting having discussed sex-related matters was low (between 8% and 38%), the proportion reporting communication about contraceptives was even lower with no more than 10% reporting such communication (with the exception of females in Uganda) [41]. Finally, a cross-sectional study from Nigeria found that 30% of adolescents reported seeking information about 'sexual matters' from their parents. This study also found that there was a significant relationship between source of information and sexual experience, for instance, a greater proportion of adolescents (55%) who received sexuality information from peers were sexually experienced compared with 34% who sought information from parents and other sources (p < 0.01) [47].

However, comparison across studies is difficult since questions concerning frequency of communication were posed differently in the various studies and in some papers, the formulation of the question was not reported. Two papers from a comparative study among South African and Tanzanian students measured frequency of communication by providing 4 response options (1 = never; 2 = hardly ever; 3 = sometimes; 4 = a lot; and 5 = all the time), with 1 and 2 dichotomized to no communication or 'silence' and 3-5 coded as having communicated [32,33]. Other studies used a similar scale, but with four points where 1 = never and 4 = more than four times [37,38]. One study used discussion about sexual abstinence with either parent in the past 12 months as a proxy measure for parent-child communication about sex [39] while another similarly used discussion about sex or sexuality in the household with parents, guardians or 'other household members' during the last 12 months as an indicator for parent-child communication [48]. One study in Nigeria constructed a 12-point Family Life Communication Scale (FLCS), where discussion of a particular topic area could receive a maximum of 2 points if either parent had ever initiated discussion about sexuality their adolescent, 1 point if someone other than the parent had initiated such a discussion and no points if it was never discussed [42]. The CHAMP intervention study in South Africa provided response options ranging from 'we talk about this a lot' to ' we never talk about this' [49]. Adopting a more open-ended approach, two other studies measures of parent-child communication which queried 'which types of people had talked to the adolescent about sex-related matters' and about 'the types of people or other sources from which the adolescent received information about contraceptive methods' [41,45]. Finally, another study asked students if they had 'ever talked about HIV/AIDS with parents or other adults in the family', dichotomizing the response options to 'yes' and 'no' [34], while a similar yes/no dichotomization took place in another study [40]. The fact that several of the studies include terms such as guardians, other adults, and household members when inquiring about communication reflects the role of the extended family in the sexual socialization of young people in SSA. However, this makes it difficult to ascertain precisely who communicated with the child. These examples highlight the range of sensitivity employed in measuring frequency of discussion in the various studies.

One qualitative study sought to determine frequency of discussions, but found that it was difficult for participants to tell how often they discussed, while the few who were able to make an estimation reported a range from once in a day to once a month or several months [50]. These findings points to the potential for recall bias in the quantitative studies.

#### Triggers for parent-child sexuality communication

Only one study reviewed investigated triggers for discussions about HIV/AIDS, and it was reported that parents frequently used examples of relatives who had died of AIDS to initiate a discussion and to reiterate the severity of the disease. Other triggers for discussion reported by parents in this study were radio programs, flyers, parental perceptions of risky sexual behavior, or seeing someone they believed was HIV positive, for instance due to thinness [50].

#### Factors associated with sexuality communication

Identifying factors associated with and predictors of sexuality communication is an important precursor to the development of programs and interventions to improve parent-child sexuality communication. A total of 12 studies reported data relating to this topic. One comparative study of parent-child communication in the United States and Kenya found that in both countries, the odds of communication about HIV/AIDS taking place were associated with parental perceptions that their child was ready to learn about sexuality, whether or not they had received information to educate their child about sex, and if they had greater sexual communication responsiveness. Sexual communication responsiveness was defined as the skill, comfort & confidence to communicate about sexuality [40]. Regarding the second factor, having received information to educate their child about sex, the authors note that due to the wording of the item, it is not clear whether this particular finding relates to motivation or knowledge.

Another study found that young people living in rural areas reported more frequent communication about HIV/AIDS with both mothers and fathers than those living in urban areas. In addition, attending school and having a higher socioeconomic status were found to be associated with more frequent communication with parents [38]. In a multi-site study conducted in South Africa and Tanzania, higher socio-economic status was similarly found to be significantly associated with more frequent communication with parents in both of the South African sites, but not in Tanzania [32]. Consistent with these findings, higher socio-economic status was also reported to increase frequency of parental communication in Nigeria [42]. This study also found that positive perceptions of the parental role and responsibility in family life education was associated with increased frequency of communication, as was greater time spent at home by parents.

In studies conducted in Nigeria and Kenya, it was found that the education level of the parents was associated with whether or not sexuality and HIV/AIDS had been discussed, with those having a higher level of education most likely to have had communication with their children [35,40,42,43]. Parental marital status was also found to play a role in Kenya. In terms of religious affiliation, this was found to be a significant predictor of experiencing silence in one site in South Africa (Mankweng), with Catholics and other affiliations significantly more likely to report silence than Protestants [32]. Age was not found to be associated with communication about HIV/AIDS in one of the studies [38], but this was not consistent with others studies in the review. For instance, in the multi-site study, it was found that students who communicated about HIV and sex with parents were more likely to be older in Tanzania and one of the South African sites (Mankweng), while age was not associated with silence in the Cape Town site [32,33]. In a study conducted in Ghana, findings showed that sexuality communication is more common among older respondents than younger ones [45]. In terms of relationship status, one multi-site study found that those more likely to communicate with either parents or teachers were those who had a boy/girlfriend and who had used alcohol [33]. In addition, another study found that family size was associated with more frequent communication about sexuality with families of 5 or less more likely to discuss [35].

Gender differences were noted by most studies in terms of frequency of communication, although the findings are mixed. For instance, in Nigeria and Ghana, it was found that significantly higher proportions of female students reported family communication about sexuality than males (81% versus 63%, and 46% verus 28% for females and males respectively in two studies conducted in Ghana; 86% versus 76% for females and males in a study from Nigeria) [34,35,45,47]. In a multi-site study conducted in South Africa and Tanzania it was found that after controlling for other socio-demographic variables in South Africa, a greater proportion of males reported silence from their parents concerning HIV/AIDS, abstinence and condoms compared to females, while the reverse was found in Tanzania where a higher proportion of females reported non-communication with parents on these issues compared to males [32]. However, this finding is in contrast to another study which used the same population of students and which found that more girls than boys reported they communicated with parents about HIV and sex. This study however, adopted a longitudinal approach rather than using baseline data only, and restricted its analyses to virgins in the sample [33]. This is consistent with another multi-country study conducted in Burkina Faso, Ghana, Malawi and Uganda which found that males in three countries were less likely than females to report parental communication about sex [41]. Several studies reported that the mother was the most frequent communicator or initiator of sexuality discussions [35,50]. However, it was found that mothers did not fully exploit this advantage to have more open discussions with their children; rather, they continued to communicate through the use of warnings and threats [50].

One of the factors associated with sexuality communication relates to timing and parental perceptions that their child has had his/her sexual debut. One study in Tanzania found that parents tended to wait until their daughters were in secondary school to initiate discussions about sexuality, due to the assumption and expectation that those still in primary school were not sexually active [50]. Consequently, there was reported to be increased secrecy in sexual relationships and also increased difficulty in accessing contraceptives for fear of being found to be sexually active. A study conducted among women in Nigeria reported that 41% believed sexuality education should commence between the ages of 6-10 years, whilst 32% favored starting discussions with children between the ages of 11-15 years [43]. Others however based the decision to initiate a discussion related to sexuality on observations of changes in behavior which are perceived to indicate the onset of sexual activity.

#### Communication style and tone of discussions

Several studies in the review suggest that one of the most substantial challenges to positive and effective parent-child sexuality communication relates to the communication style and tone of discussions. However, only 4 studies reviewed investigated this topic. As one study in Ghana found, communication often takes the form of instruction rather than dialogue. The study also found that communication is frequently gendered, for instance with advice given to sons to be careful, while warnings are given to girls to avoid sexual encounters with boys [45]. Similarly, a study of Nigerian parents found that parents preferred to be the initiators and dominators of discussions and perceived that if their child did so, it meant they were sexually active or planning to be. Parents in this study reportedly used imprecise terminology and tended to employ warnings and threats about sexuality rather than engage their child in dialogue [44]. A recent ethnographic study conducted in rural Tanzania found that sexuality communication was most often unidirectional, initiated by parents and took the form of warnings or threats or sometimes gossip [50]. The consequences of premarital sex in particular were described as being the most specific and concrete. However, some parents reportedly aimed rather to emphasize the benefits of education and focus on a future oriented perspective in discussions. In rural South Africa, similar findings were reported concerning the style of communication which tended to be perceived as being judgmental, proscriptive, and negative towards young people's sexuality [48]. According to the respondents in this study, it was not the act of discussing sexuality with parents that young people were opposed to per se; rather, it was the style that was focused on and identified as a barrier to discussion.

Since some parents reportedly perceive discussions about sexuality between parent and child as being shameful, immoral or inappropriate given the sensitive nature of sexuality, one study conducted in Tanzania among young people aged 14-24 years and their parents reported that euphemisms were commonly employed to discuss sex rather than explicit terminology [50]. The ability to openly discuss sexuality was however found to be moderated by parent's level of education, similar to the finding that frequency of discussion is related to parental level of education [50]. In terms of quantitative data, one study reviewed asked respondents whether they felt 'free/open' in discussing topics related to sex, and what topics were covered [48].

#### Sexuality communication preferences

In terms of preferences, findings from four studies reviewed which investigated this topic found that young people prefer sexuality communication to take place with the parent of the same sex. The South Africa-Tanzania (SATZ) study conducted among young people aged 11-17 years reported that overall, 44% of participants preferred to communicate with mothers about sexuality, while 15% preferred fathers [32]. Mothers were the preferred communication partner by the majority of female adolescents in both Tanzania and South Africa. In Cape Town, 31% preferred discussing with mothers, and 22% stated a preference for fathers, while in the other two sites, a greater proportion of males preferred discussing with fathers in comparison to mothers (47% and 27% in Dar es Salaam and Mankweng, respectively). Another study in Tanzania found that among in- and out-of-school males, 11% and 10% respectively selected fathers as a preferred partner for communicating about sexuality [38]. Among in- and out-of-school females, the study found that mothers were the first choice by both groups, with 44% and 37% of in- and out-of-school females reporting mothers as the preferred sexuality communicator, respectively. From a parental perspective, a study of Nigerian mothers and fathers parents found that they also preferred same sex discussions with their children [44].

In spite of these findings which tend to favor mothers as the preferred sexuality communicators, qualitative findings suggest that mothers are not always perceived in a positive light. For instance, focus group findings from young people in Ghana aged 14-17 years classified mothers into four categories: those who are approachable (and in the minority), those who tended to brush off questions and suggest that such discussions should take place with someone else (such as another family member), those who reacted by shouting when sexuality discussions are initiated, and those who seemed to have difficulty maintaining confidentiality and were subsequently labeled 'gossipers' [45]. In these focus group discussions, it was also found that fathers were often labeled as 'tyrants' who lacked listening skills and were prone to threaten or take action against their children's friends of the opposite sex.

#### Barriers to sexuality communication

In discussing barriers to open and frank communication about sexuality between caregivers and children, several studies mention that at least in the past, it was not considered normative in many African settings for such discussions to take place between parents and offspring. A total of 7 studies presented data on barriers to parent-child sexuality communication. From a parental perspective, a number of studies reported on barriers to sexuality communication. An article based on data collected in 1996 and 2003 in Kenya investigated the reason why educated mothers do not give 'meaningful' sex education to their daughters and identified a host of socio-cultural and religious barriers to sexuality communication [29]. In particular, four factors hindering meaningful communication are discussed, including: 1) residual traditional barriers, 2) inhibitions due to European Christianity, 3) reliance on sex education books, and 4) reliance on school teachers. The majority of mothers interviewed for the study indicated that they themselves had not received pubertal or sex education from their own mothers and were thus inhibited to providing it to their own daughters due to residual barriers which fostered a sense of unease and avoidance concerning parent-child sexuality communication. European Christianity is also discussed as influencing the type of language used to discuss sexuality, to explain for instance why metaphors and other linguistic devices are used to avoid direct communication and precise terminology which is perceived as being 'dirty'. The third and fourth factors are interrelated and point to mothers' reliance on books and school teachers to provide sex education to their daughters, in spite of the fact that school teachers have undergone the same socialization process and reportedly experience the same discomfort as mothers in discussing sexuality [29].

Also from a parental perspective, a study conducted in Kenya found that 38% of parents thought that talking about sexuality encourages sex, however the study's hypothesis that parental attitudes in this regard would influence communication were not supported [40]. The belief that discussing sexuality with children will lead to early sexual experimentation is documented by several other studies [36,44,50]. Finally, 61% of parents of 10-12 year old children in Kenya thought that they were too young to learn about sex [40]. These findings highlight a range of barriers perceived both by adults and young people to communicating about sex-related topics. Another study conducted in Nigeria found that low levels of communication were related to parental perceptions of their child's readiness or maturity, the assumption that their child would have heard about these issues elsewhere, that discussions of contraception for instance should be restricted to married people, and the often cited concern that such discussions may 'corrupt' young people or encourage early experimentation [42].

Studies also investigated young people's perspectives on barriers to sexuality communication with their parents. A number of studies identified parental lack of knowledge of sexual and reproductive health as a barrier to communicating with their children. For instance, one study in Nigeria found that 64% of secondary school students perceived their mothers as lacking sufficient knowledge, while 87% thought fathers lacked knowledge [36]. In identifying other barriers to communication, this study found that 62.3% thought that their parents are too preoccupied to talk about sex, while 59% believed their parents would argue if they were to talk about sex. In addition, 30% thought their mother would think they were interested in experimenting with sex if they were to talk about it, whilst 69% believed their father would get this impression.

Focus group data from Ghana show that young people are reluctant to discuss sexuality with their parents since they tend to prefer to discuss these issues with their friends, because they feel shy, and also because they may fear physical punishment for discussing sexuality [45]. The fear of physical punishment or blame was even said to deter reporting to parents that unconsensual or unwanted sex had occurred.

### Behavioral outcomes associated with parent-child sexuality communication

#### Associations with sexual intentions, behavior and contraceptive use

The second topic we reviewed focused on studies which investigated behavioral outcomes associated with parent-child sexuality communication. We identified 6 studies which focused on abstinence and delayed sexual debut, and 3 studies which focused on contraceptive use. In terms of parental sexuality communication and odds of sexual activity, the findings from the multi-country study were inconsistent across countries. Among Malawian males and Ugandan females only, sexuality communication was associated with increased odds of having had sex in the last 12 months [41]. A cross-sectional study from the Ivory Coast found that parent-child communication in the last 12 months about abstinence was associated with an earlier sexual transition among boys, whereas it was associated with a delayed sexual debut or primary sexual abstinence, as well as secondary sexual abstinence (defined as abstinence in the last 6 months) and a reduction in number of sexual partners among girls [39]. A cross-sectional study in Nigeria found that those who reported having been 'instructed' by their mothers about sexual matters before puberty were more likely to have had their sexual debut than those who had not received the same instruction [47]. However, it is unclear from the data presented whether or not this was true for both boys and girls in the sample. In Ghana, one cross-sectional study found that communication about sex-related topics with fathers only was negatively associated with having had sex in the last 12 months among boys, but positively associated with sex in the last 12 months among girls [45]. Other findings from cross-sectional studies in Ghana suggest mixed findings on this issue, one of which did not find a similar association [34] and another which found that communication with family members about avoiding sex was associated with decreased odds of having had sex among males [46]. Finally, a study which investigated sexuality communication and timing of sexual debut in Tanzania found that while communication with teachers about HIV and sex was associated with a delayed sexual debut, parental communication was not after adjusting for age and conducting multivariate logistic regression [33].

Based on the studies reviewed here, the relationship between sexuality communication and contraceptive use is unclear. The study conducted in 4 African countries found that parent-child sexuality communication was positively associated with contraceptive use for Ghanaian females and for Ugandan females and males [41]. Another study conducted in Ghana found that communication about contraceptive use was associated with an increased likelihood of being sexually experienced among both sexes [46]. The study also found that the association between communication about sex and contraception and condom use was overall weak, except where it concerned consistent condom use with the last partner among males [46]. However, the same study also reported the puzzling finding that communication about contraceptives was associated with a decreased likelihood of condom use at first sex among males. Evidence from a cross-sectional study conducted in Ghana found that communication about HIV/AIDS between students and parents or other family members increased the odds of using a condom at last sexual intercourse [34]. Although this finding is promising, due to the wording of the question, it is not possible to determine who the communication took place with and what role gender may play in moderating this effect.

### Intervention studies focused on improving parent-child sexuality communication

There are few examples of interventions with an explicit focus on promoting and improving sexuality communication between parents and offspring in sub-Saharan Africa. However, results from the four intervention studies reviewed here are promising. One recent intervention called IMAGE (Intervention with Microfinance for AIDS and Gender Equity) in rural South Africa designed to reduce levels of HIV and intimate partner violence also included a component which aimed to promote communication between adults and young people [48]. The quantitative and qualitative findings from this study indicate that women who participated in intervention activities reported significantly more frequent discussion about sexuality with children than women in the control group. Moreover, both intervention participants and young people reported that as a result of their participation, the content of the discussions became more specific and concrete about risk reduction strategies rather than vague messages or admonitions. Women who participated in the intervention felt that they had developed a skill set which enabled them to be more comfortable in discussing sexuality. Several other positive benefits were reported by intervention participants that are worth mentioning. It was reported that as women's awareness and skills grew through their participation, they began to feel a heightened sense of responsibility and obligation to share their knowledge with others in their community for the benefit of young people. Furthermore, a need for change in terms of cultural norms and expectations which impede parent-child sexuality communication was also recognized. However gender norms were more difficult to reconcile with several reporting difficulty in discussing sexuality with boys and opting for indirect strategies to convey risk reduction messages.

The quantitative data from this study also suggests positive intervention results, with an increase in the frequency and perceived comfort level of intervention participants to discuss HIV and other sex-related topics in comparison to the matched women in the control group [48]. This was the only study identified for this review which compared responses of parents and young people. The findings indicate that fewer young people reported having communicated about sexuality with parents in comparison to parental reports (51% vs. 80%). However, as the authors note, the findings are not exactly comparable since young people were asked about communication with *either *parent and/or guardians, whilst mothers were asked about communication with their own *and/or *their friends' children. Young people residing in intervention households in this study reported higher levels of communication compared with a matched cohort in control villages; however the effect was not statistically significant when cluster analysis was applied to the data.

Another family-based HIV prevention intervention in South Africa called CHAMP (Collaborative HIV/AIDS and Adolescent Mental Health Programme) has been adapted from the United States [30], with positive results reported among parents [49]. This intervention attempted to strengthen what it identified as key family processes which influence youth risk-taking behavior such as parental monitoring, discipline effectiveness, and parent/caregiver and youth frequency and comfort in communicating about sensitive topics. In the South African context, the intervention consisted of a series of 10 manual-based sessions delivered to families with children between the ages of 10 and 11 years, with a qualitative process evaluation of the intervention at two year follow up of the same cohort [51]. A wide range of topics were included in the manual such as parenting styles, monitoring of children, improving communication between parent and child on sensitive topics such as puberty, among others. The intervention was described as utilizing Freireian principles of participatory adult education and critical consciousness through small group experiential learning. In particular, an open-ended participatory cartoon narrative was used to engage families, with a workbook with assignments to take home to involve other family members.

In addition to other measures such as AIDS knowledge, social networks and stigma, the study assessed parental communication styles and topics that parents have difficulty talking about with their children. In terms of parenting communication styles, findings showed that the intervention group made a significant shift from engaging passive aggressive and manipulative communication styles to more assertive styles in relation to comparison group. The intervention group also showed significant improvement in their ability to discuss sensitive topics with their children in comparison to control group. Frequency of discussion was also reported to improve in the intervention group, for instance discussion about puberty increased from 55% to 69%, whilst discussion about sex which was ranked most difficult to talk about improved from 55% to 73% post intervention [49]. Findings from the qualitative follow-up evaluation of the intervention reported positive findings in terms of empowerment at the individual level (both parental and as a woman) and the collective level, enhanced perceptions of social support and social capital [51].

The CHAMP intervention has also been subsequently tested and evaluated among black South Africans [52]. This intervention also reported positive findings, however with greater intervention effects observed on caregivers than on youth. For instance, caregiver findings demonstrated significant intervention group differences in comparison to control with regard to caregiver monitoring and control of their children's whereabouts and behavior. In addition, the intervention group reported increased frequency and comfort discussing HIV/AIDS and sexuality with their children, among other outcomes. With regard to youth, findings showed an increase in HIV knowledge in the intervention group compared to the control, as well as lower levels of stigma, with more limited effects on communication.

Finally, the Families Matter! Program, also adapted from a US to Kenyan context has recently been evaluated to assess intervention effect on parenting practices and parent-child communication [53]. The objective of the community-based program is to improve protective parenting skills, knowledge, comfort and confidence of parents of children aged 9-12 years so that they can communicate about sexual reduction prior to the onset of sexual activity. Participatory learning strategies are utilized in a series of small group sessions delivered by trained facilitators. In the final session, children join and participate in a guided communication exercise. Evaluation of the program focused on 3 measures: parental attitudes towards sexuality education and whether children had ever asked their parent about a sex issue, parenting practices (comprised of parent-child relationship, positive reinforcement and monitoring) and parental responsiveness (conceptualized as parents' skill, comfort and confidence to communicate about sexuality with their child) [53]. Positive intervention effects at follow-up were found on all parenting measures except parents' report of the parent-child relationship, which the authors' note was already high at baseline with little room for improvement [53]. The intervention also positively impacted frequency of parent-child sexuality communication from 17% of children reporting having asked their parent a question about sexuality at baseline to 38% at 12 months follow-up, and 14% of parents reporting being asked a question about sexuality from their child at baseline, to 50% at follow-up [53]. In addition to these positive intervention effects, the study also found that the program was well received by parents who found it beneficial in terms of skill and confidence development, and also changed their overall attitude towards sexuality education including the belief that communicating about sexuality leads to early sexual experimentation.

## Discussion

The number of studies aiming to understand and improve parent-child sexuality communication in SSA is steadily increasing. Although this is a promising finding in itself, it is evident from this review that there is substantial variation in measurement and methods of investigating sexuality communication, effectively limiting the conclusions that can be drawn. Indeed, given the heterogeneity of the measures employed in the studies included in this review, it is clear that there is a need to standardize communication measures so that comparisons can be made across studies.

The findings of the review demonstrate that the frequency of discussions about sexuality between parents and offspring varies markedly across contexts. Important information concerning the myriad factors associated with parent-child sexuality communication can be gleaned from the findings of this review, although the consistency in study findings suggests the need for further research on this topic. A range of socio-demographic characteristics were identified as being associated with parent-child sexuality communication by the studies, including sex, age, urban or rural residence, socio-economic status, school attendance, parental level of education, religious affiliation and other household characteristics such as family size and marital status of the parents. Inconsistencies across studies likely point to contextual or methodological differences.

Aside from demographics, other factors which may be more amenable to change through future interventions and programs were identified. For instance, the findings from a comparative study of factors associated with parent-child communication about HIV/AIDS in the United States and Kenya identified the same three factors, suggesting robustness across contexts. In particular, the findings of this study indicate that parental perceptions of readiness of their child to learn about sexuality are highly influential, as are parental acquisition of information to assist them in educating their child, and finally, having a high level of 'sexual communication responsiveness'. Findings of several studies reiterated the importance of timeliness in sexuality discussions, of particular concern since programs and interventions have been found to be most effective when they target young people prior to their sexual debut. Thus, understanding how parents assess whether or not their child has had their sexual debut and conveying to them that discussions need to take place before they believe their child has had their sexual transition are important aspects to be considered when attempting to improve parent-child sexuality communication. Future research on outcome expectancies seems particularly important, given widespread concerns that sexuality discussions will lead to early experimentation, and interventions to address this misconception are also necessary to encouraging proactive communication.

A number of barriers to parent-child sexuality communication were also identified by the review, which should be taken into consideration in the development of interventions and programs. For instance, as mentioned above, lack of parental knowledge was reportedly a barrier, as perceived by parents and young people alike. This suggests that programmatic efforts need to target not only parental knowledge, but their self-efficacy and comfort to communicate with their children about sexuality. Skills based programs which incorporate role plays may be one way to improve parental communication skills. In addition, since several studies in the review found that communication tends to be uni-directional, authoritarian and negative towards sexuality, role play may assist parents in efforts to engage their children in a more dialogical, positive approach. Given the negative perceptions of parents that emerged from the qualitative studies on parental communication, it is clear that fostering the development of communication skills for parents is of importance. The cluster of attributes defined as 'parental responsiveness', including being skilled, comfortable and confident, may impact the quality of parent-child sexuality communication. Since quality may subsequently impact important outcomes such as knowledge, attitudes, intentions and behaviors, these interrelated issues need to be addressed more systematically in future studies.

In addition, the studies also pointed to the social and cultural norms which demarcate the boundaries surrounding sexuality communication with children in SSA which need to be addressed in efforts to promote and improve communication. Historical factors also play a role in shaping sexuality communication as one study in Kenya found, with residual traditional barriers and inhibitions related to European Christianity inhibiting open and direct communication. These findings demonstrate that efforts need to be multi-level and directed at the community level to increase awareness of the importance of parent-child sexuality communication.

In terms of the impact of communication on sexual intentions, behavior and contraceptive use, findings from this review are inconclusive and this is a topic of importance for future studies, particularly since a number of studies identified an association between parental communication about sexuality and sexual activity. Findings suggest a differential effect for boys and girls, and again, the issue of timing likely plays a key role in this regard.

There is a paucity of family-based interventions or those which target parent-child sexuality communication in SSA which have been rigorously evaluated, but the available findings are promising. The findings reviewed here suggest that if parents are given support to develop the attributes comprising 'parental responsiveness', they can and will communicate with their children about HIV/AIDS and sexuality. The interventions reviewed here demonstrate that it is possible to increase frequency of discussion about sensitive topics and improve the comfort level of parents, while also improving the content of the discussions to the extent that they are more explicit and less vague. The findings also point to the possibility of raising awareness of social and cultural norms which were hindering sexuality communication with the aim to begin to challenge these norms. However, as one particularly critical study highlighted, the theoretical, conceptual and methodological bases for advocating parent-child sexuality communication require careful examination and consideration in the development of such programs and interventions.

This review of parent-child sexuality communication in sub-Saharan Africa points to several gaps in the extant literature. Firstly, since the timing of sexuality discussions may impact the effectiveness of communication and since it has been identified as a particularly contentious issue, further research is needed to fully understand parental concerns and how these can be addressed in interventions and programs aiming to promote sexuality communication. Closely linked with timing are the issues of content and tone of discussions. As the findings in this review indicate, parents frequently favor indirect communication strategies and when communication is more direct it often comes in the form of warnings, admonitions and lectures. More qualitative data are needed to understand more fully how parents communicate with their children about sexuality and the strategies they employ. An additional gap identified by this review is the lack of information available about perceptions of quality of parent-child sexuality communication. Also limiting our understanding of parent-child sexuality communication, the qualitative process studies we reviewed focused primarily on mothers, leaving a major gap in our understanding of the role that fathers play in the parenting process and sexual socialization of children in sub-Saharan Africa. Further studies on this topic would be particularly useful to gain a more in-depth understanding of their role and thus determine how interventions can best strengthen their involvement. In addition, several studies in the review mention that future studies should investigate dimensions of parenting, including style, monitoring and control, attitudes, connectedness and communication, all of which may impact sexuality communication and which clearly merit further investigation. Finally, greater understanding is also needed of how social change is influencing the family unit, including the effects of increasing urbanization in Africa especially as it relates to the move to more nuclear families as opposed to living with the extended family and subsequently impacting communication. It is likely that this varies in urban and rural settings and more data are needed to understand how traditional practices and modes of sexual socialization are shifting.

There are additional limitations to consider when interpreting the findings of this review. Since we did not include studies which were not peer reviewed, we have not tapped into the grey literature on this subject, nor have we included theses and dissertations which may contribute relevant findings. In addition, although we did not identify any studies in languages other than English, there is a possibility that we may have missed studies written in other languages. In addition, in focusing on parent or caregiver-child sexuality communication, we have not reviewed studies related to issues such as parental control and monitoring, which is a topic receiving growing interest [54,55]. Nor have we included studies which focus broadly on sexual socialization of children in African countries and studies which have more limited discussions of parental involvement. Finally, we also did not include studies which investigate other influential family members such as aunts. For instance, several studies have shown that aunt's played a key role in the sexual socialization of young girls [20,56,57].

It is also important to consider the methodological limitations of the studies themselves. The findings of the review are limited by the cross-sectional nature of the majority of the quantitative studies, which are limited in terms of establishing causality. In addition, several studies utilized convenience samples and therefore cannot be considered representative. Also, given the nature of self-report data, study results may have been biased. Finally, the fact that the majority of studies utilized only one perspective (either parent or child) may also have biased the findings of this review.

## Conclusions

Whilst the number of studies investigating parent-child sexuality communication in sub-Saharan Africa is increasing, there is a need for future research employing both qualitative and quantitative methods which elucidates the factors influencing communication, including skills and intentions, as well as barriers and facilitators. This is particularly important given the tentative but promising findings of this review that parents will talk to their children about sexuality if given the appropriate support..

## Competing interests

The authors declare that they have no competing interests.

## Authors' contributions

SB conceived the study and drafted the manuscript. LKM and WMM participated in drafting the manuscript. All authors read and approved the final manuscript.
